# Role of TrkB expression in rat adrenal gland during acute immobilization stress

**DOI:** 10.1111/jnc.12030

**Published:** 2012-10-25

**Authors:** Yusuke Kondo, Masahiro To, Juri Saruta, Takashi Hayashi, Hiroki Sugiyama, Keiichi Tsukinoki

**Affiliations:** Department of Environmental Pathology and Research Institute of Salivary Gland and Health Medicine, Graduate School of Kanagawa Dental CollegeYokosuka, Japan

**Keywords:** acute immobilization stress, adrenal medulla, agonist antibody, brain-derived neurotrophic factor, catecholamine, tyrosine receptor kinase B

## Abstract

Expression of tyrosine receptor kinase B (TrkB), a receptor for brain-derived neurotrophic factor (BDNF), is markedly elevated in the adrenal medulla during immobilization stress. Catecholamine release was confirmed *in vitro* by stimulating chromaffin cells with recombinant BDNF. We investigated the role of TrkB and the localization of BDNF in the adrenal gland during immobilization stress for 60 min. Blood catecholamine levels increased after stimulation with TrkB expressed in the adrenal medulla during 60-min stress; however, blood catecholamine levels did not increase in adrenalectomized rats. Furthermore, expression of BDNF mRNA and protein was detected in the adrenal medulla during 60-min stress. Similarly, in rats undergoing sympathetic nerve block with propranolol, BDNF mRNA and protein were detected in the adrenal medulla during 60-min stress. These results suggest that signal transduction of TrkB in the adrenal medulla evokes catecholamine release. In addition, catecholamine release was evoked by both the hypothalamic–pituitary–adrenal axis and autocrine signaling by BDNF in the adrenal gland. BDNF–TrkB interaction may play a role in a positive feedback loop in the adrenal medulla during immobilization stress.

Brain-derived neurotrophic factor (BDNF) belongs to the neurotrophin family, which includes nerve growth factor, as well as neurotrophin-3, -4/5, -6, and 7 (Saruta *et al*. 2010c). BDNF is mainly expressed in the peripheral nervous system and CNS, and plays important roles in the survival, differentiation, maintenance, and protection of neural cells (Cirulli and Alleva 2009). BDNF binds to tyrosine receptor kinase B (TrkB) and p75 neurotrophin receptor (p75) and has high affinity for TrkB (Numakawa *et al*. 2010). TrkB is expressed in both the CNS and peripheral tissues, including the adrenal gland, Kupffer cells in the liver and salivary glands (García-Suárez *et al*. 2006; Kondo *et al*. 2010; Saruta *et al*. 2010a). BDNF binding to TrkB induces receptor dimerization, phosphorylation, and activation of the intracellular tyrosine kinase domain. These events initiate several complex intracellular signal transduction cascades, which subsequently induce biological responses (Tapia-Arancibia *et al*. 2004). However, in peripheral tissue, the role of interaction between BDNF and TrkB is poorly understood.

Brain-derived neurotrophic factor is involved in neuropathies such as depression (Karege *et al*. 2005), schizophrenia (Tan *et al*. 2005), Alzheimer's disease (Michalski and Fahnestock 2003), and Parkinson's disease (Parain *et al*. 1999). Alteration of BDNF content in the central nervous system reportedly plays an important role in the pathogenesis of neural diseases (Lommatzsch *et al*. 2005; Tsukinoki *et al*. 2007). Furthermore, BDNF–TrkB interaction is involved in stress, as acute immobilization stress decreases BDNF contents in the hippocampus (Ueyama *et al*. 1997), and more interestingly, decreases TrkB contents in the pituitary gland (Givalois *et al*. 2001). Our previous studies have shown that expression of BDNF increases in rat salivary glands under acute stress conditions (Tsukinoki *et al*. 2006), and biting behavior increases BDNF levels in the hippocampus (Lee *et al*. 2008). These results suggest that BDNF is up-regulated by stress in the CNS and peripheral tissues, and that it plays a role in the bioactive response to stress.

*In vivo*, stress response is induced by activation of the hypothalamic–pituitary–adrenal axis and the sympathetic–adrenal–medullary axis (SAM axis) (Harris 1955; Chen and Miller 2007). Activation of the SAM axis through the adrenal medulla is known to affect ‘fight and flight’, as proposed by Cannon (Cannon 1929; Segerstrom and Miller 2004). The adrenal medulla is differentiated into endocrine organs during the development of the sympathetic ganglia, and plays an important role in the sympathetic nervous system (Segerstrom and Miller 2004)**.** In the response to stress, catecholamine release from the adrenal medulla causes increases in the metabolic rate of the whole body by binding to alpha and beta receptors (Guyton and Hall 1996; Barrett *et al*. 2009). For example, activation of alpha1 receptors has a vasoconstrictive effect, activation of beta1 receptors leads to increased cardiac output, and in liver, activation of alpha and/or beta receptors induces hyperglycemia via glycogen degradation (Keiver and Hochachka 1991; Katz *et al*. 1993; Sharara-Chami *et al*. 2012).

Our previous studies have shown that catecholamines are released from PC12 cells (derived from chromaffin cells) stimulated with recombinant BDNF *in vitro*. In addition, we demonstrated *in vivo* that chromaffin cells of the adrenal medulla showed significantly increased TrkB mRNA and protein levels after 60 min of stress (Kondo *et al*. 2010). However, the role of TrkB expressed in the adrenal medulla after 60 min of acute immobilization stress remains unclear, particularly with regard to its action on catecholamine release *in vivo*. Therefore, we investigated the role of the TrkB expressed in the adrenal medulla, as well as the expression and localization of BDNF in the adrenal gland under acute immobilization stress for 60 min. Furthermore, we examined the BDNF–TrkB interaction against stress response in the adrenal medulla.

## Materials and methods

### Characterization of anti-TrkB (clone 47/TrkB)

First, we confirmed that the TrkB receptor was activated by mouse monoclonal anti-TrkB (clone 47/TrkB) (BD Biosciences, San Jose, CA, USA) using PC12 cells. The purpose of this experiment was to distinguish from the role of TrkB activated by endogenous BDNF *in vivo*, thereby clarifying the role of TrkB. PC12 cells derived from chromaffin cells of the rat adrenal medulla (DS Pharma Biomedical Co., Ltd., Osaka, Japan) were seeded on collagen IV-coated culture dishes (BD Biosciences) and were cultured in RPMI1640 (Invitrogen Corp., Carlsbad, CA, USA) supplemented with 10% fetal bovine serum (Thermo Fisher Scientific Inc., Waltham, MA, USA) at 37°C under 5% CO_2_ (Kondo *et al*. 2010).

PC12 cells were stimulated with culture medium containing 30 μg/mL anti-TrkB (clone 47/TrkB) (agonist group). Trk signaling of PC12 cells was blocked by addition of 10 μg/mL K252a (Alomone Labs, Ltd., Jerusalem, Israel), which is an inhibitor of tyrosine phosphorylation of Trk receptors, to the culture medium for 5 min. Cells were then stimulated with fresh medium containing 30 μg/mL anti-TrkB (clone 47/TrkB) for 5 min (K252a+agonist group). For control experiments, PC12 cells were incubated in medium without serum (control group). After treatment, catecholamines in the medium, including adrenaline, noradrenaline, and dopamine, were analyzed after fractionation using HPLC.

### Animals

Nine-week-old male Sprague–Dawley rats (Japan SLC, Inc., Shizuoka, Japan) were used in this study. Rats were housed in groups of six animals per cage in a room maintained under standardized light (12 : 12-h light-dark cycle) and temperature (22 ± 3°C) conditions. The animals had free access to food pellets and tap water. To avoid diurnal variations in TrkB expression, all rats were killed between 07:00 hours and 11:00 hours.

## Experimental procedures

The experimental protocol used in this study was reviewed and approved by the Ethics Committee for Animal Experiments of Kanagawa Dental College and was carried out in accordance with the Guidelines for Animal Experimentation of Kanagawa Dental College.

Rats were divided into four groups of four rats each: control, stress, stress+agonist, and stress (adrenalectomy)+agonist. The control group was not exposed to immobilization stress. Non-control groups were immobilized to produce acute stress according to a well-established protocol (Tsukinoki *et al*. 2006; Lee *et al*. 2008). Briefly, rats were fixed with a leather belt onto a wooden board (18 × 25 cm) in a supine position, and all legs were fixed at an angle of 45° to the body midline with adhesive tape. Rats were exposed to immobilization stress for 60 min, as this was the time at which the expression of TrkB m RNA and protein peaked in adrenal medulla in our previous studies (Kondo *et al*. 2010). Rats in the stress group were exposed to the above condition only, while TrkB agonist antibody (anti-TrkB; clone 47/TrkB) was administered via tail vein injection in the stress+agonist group and stress (adrenalectomy)+agonist group under deep anesthesia with sevoflurane after 60-min immobilization stress.

The adrenal glands were removed bilaterally (adrenalectomy) or a sham operation performed was under sodium pentobarbital anesthesia (65 mg/kg i.p.) at 3 weeks prior to immobilization stress experiments (age, 6 weeks; Smith *et al*. 1995; Saruta *et al*. 2010b). A 2-cm skin incision was made in the abdomen, the bilateral adrenal glands were isolated from the surrounding visceral fat tissue, a ligature was placed around the supplying vessels, and the adrenal glands were removed.

Rats in the stress group were killed by deep anesthesia with sevoflurane after immobilization stress for 60 min. Rats in the stress+agonist and stress (adrenalectomy)+agonist groups were killed by deep anesthesia with sevoflurane at 5 min after administration of TrkB agonist antibody (anti-TrkB; clone 47/TrkB). Subsequently, blood and adrenal glands were collected from rats in all groups. Blood was analyzed for catecholamines using HPLC, and adrenal glands were analyzed for expression of BDNF mRNA using RT-PCR.

To further investigate the role and localization of BDNF in adrenal glands, we administered propranolol as sympatholytic (5 mg/kg i.p.) to rats before 60-min immobilization stress (Koizumi *et al*. 2011). Thereafter, rats were killed by deep anesthesia with sevoflurane. Subsequently, adrenal glands were collected from rats and were analyzed for localization of BDNF mRNA and protein using *in situ* hybridization (ISH) and immunohistochemistry (IHC). In *in vivo* experiments, we compared between intact rats and sham-operated rats in each group.

### Blood sampling

After deep anesthesia of all rats at the end of experiments, blood samples were collected between 07:00 hours and 11:00 hours by cardiac puncture into Venoject II® tubes containing EDTA (Terumo, Tokyo, Japan). Tubes were immediately placed on ice, and centrifuged at 760 × *g* for 15 min at 4°C. Plasma was stored at −20°C prior to HPLC (Saruta *et al*. 2012, in press).

### Catecholamine assay of blood plasma and cell culture medium

Catecholamines in blood plasma and medium, including adrenaline, noradrenaline, and dopamine, were analyzed after fractionation by HPLC. Each sample was passed through a cation exchange pre-column to remove water soluble materials, and catecholamines were then separated on a HLC-8030 column (Toso Co., Tokyo, Japan). The fluorescence produced by diphenylethylene-diamine and potassium ferricyanide was then measured using an excitation wavelength (λ_ex_) of 355 nm and an emission wavelength (λ_em_) of 470 nm (Kondo *et al*. 2010).

### RNA extraction and cDNA synthesis

Total RNA isolation from the adrenal glands was performed using ISOGEN reagent (Nippon Gene, Toyama, Japan) in accordance with the manufacturer's instructions. RNA concentrations were determined by absorbance readings at 260 nm using a NanoDrop ND-1000 spectrophotometer (NanoDrop Technologies, Inc., Wilmington, DE, USA). Reverse transcription was performed as outlined in the instruction manual using a first-strand cDNA synthesis kit (Roche Diagnostics Ltd., Lewes, UK).

### RT-PCR of TrkB and BDNF mRNAs in adrenal gland

RNA extraction and cDNA synthesis protocols are as described above. RT-PCR was performed using TaKaRa Ex Taq (Takara Bio Inc., Shiga, Japan). The primer sequences used to amplify the TrkB receptor were forward, 5′-ATAACGGAGACTACACCCTGATGG-3′; and reverse, 5′-AGCTGACTGTTGGTGATGCC-3′ (PCR product: 505 bp) (Invitrogen) (Ohira *et al*. 2005). The primer sequences used to amplify the BDNF were forward, 5′-CAAAAGGCCAACTGAAGC-3′; and reverse, 5′-CGCCAGCCAATTCTCTTT-3′ (PCR product: 169 bp) (Invitrogen) (Aliaga *et al*. 2010). The primer sequences used to detect the internal control marker glyceraldehyde-3-phospate dehydrogenase (GAPDH) were forward, 5′-CCTTCATTGACCTCAACTAC-3′; and reverse, 5′-TTCACACCCATCACAAAC-3′ (PCR product: 306 bp) (Invitrogen) (Kondo *et al*. 2010).

PCR parameters were 94°C for 30 s, 58°C for 30 s, and 72°C for 60 s for 30 cycles, followed by a final elongation at 72°C for 5 min (Kondo *et al*. 2010). Amplified PCR products were resolved by electrophoresis on 3.0% agarose gels, stained with ethidium bromide, and photographed using LAS3000 (Fuji Film Co., Tokyo, Japan).

### Quantitative real-time PCR of BDNF mRNA in adrenal gland

Real-time PCR was performed using a LightCycler 480 System (Roche) according to the manufacturer's instructions. Reactions were performed in a 20-μL volume (BDNF: 10 pmol of each primer). Reactions with Taq DNA polymerase, nucleotides, and buffer for BDNF were performed with LightCycler FastStart DNA Master SYBR Green I (Roche). Oligonucleotide primers designed to amplify rat BDNF were specific for the coding region of exon 5. BDNF-specific primers were 5′-CAGGGGCATAGACAAAAG-3′ (forward); and 5′-CTTCCCCTTTTAATGGTC-3′ (reverse; BDNF PCR product: 167 bp; Tsukinoki *et al*. 2006; Lee *et al*. 2008; Saruta *et al*. 2010b), as designed and synthesized by Nippon Gene Research Laboratories. Real-time PCR for amplification of the rat β-actin housekeeping gene was performed using a LightCycler 480 Probes Master (Roche), 5′-CCTGTATGCCTCTGGTCGTA-3′ (forward) and 5′-CCATCTCTTGCTCGAAGTCT-3′ (reverse; β-actin PCR product: 260 bp) (Tsukinoki *et al*. 2007), in accordance with the manufacturer's instructions (Nihon Gene Research Labs Inc., Sendai, Japan). Denaturation was performed at 95°C for 10 min, after which Segment 1 (95°C for 10 s), Segment 2 (60°C for 10 s), and Segment 3 (72°C for 10 s) were repeated for 40 cycles. We performed melting analysis and agarose gel electrophoresis to confirm the specificity of the PCR products obtained using each primer pair. Gene expression is given in terms of the ratio of the copy number of BDNF mRNA to β-actin mRNA for each sample.

### ISH for BDNF mRNA in adrenal gland

Complementary RNA (cRNA) probes for BDNF mRNA were based on a 119-bp fragment corresponding to bases 2659–2778 of the rat BDNF cDNA (GenBank accession no. D10938) and were produced by *in vitro* transcription of linearized pGEM-T Easy Vectors (Promega Co., Madison, WI, USA) (Ottem *et al*. 2007). Digoxigenin (DIG)-11-UTP-labeled single-stranded cRNA probes for rat BDNF were prepared using the DIG labeling kit SP6/T7 (Roche) in accordance with the manufacturer's instructions. Procedures for ISH were as described previously (Saruta *et al*. 2005; Kondo *et al*. 2010). ISH was performed using 4 μm tissue sections. Paraffin sections were digested with 1 μg/mL proteinase K for 15 min at 37°C. Hybridization was performed at 37°C for 16 h using DIG-11-UTP-labeled single-stranded cRNA probes dissolved in hybridization medium (Wako Pure Chemical Industries, Ltd., Tokyo, Japan). After hybridization, mRNA was detected colorimetrically using a DIG-non-radioactive nucleic acid detection kit (Roche).

### IHC for BDNF protein in adrenal gland

Resected rat adrenal gland tissue samples were fixed in 4% paraformaldehyde phosphate buffer solution for 6 h and embedded in paraffin, and serial 4 μm sections were then cut and stained with hematoxylin and eosin for IHC. Immunohistochemical analysis was performed using Simple Stain Rat MAX-PO (Nichirei Biosciences Inc., Tokyo, Japan). Slides were pre-incubated in 3% H_2_O_2_ methanol for 15 min. Sections were then incubated with anti-BDNF rabbit polyclonal antibody (1 : 100; Santa Cruz Biochemistry, Santa Cruz, CA, USA) for 1 h at 24°C. After washing with phosphate-buffered saline, sections were reacted with the secondary antibody, horseradish peroxidase-labeled anti-rabbit IgG with amino acid polymer (Nichirei), for 30 min at 24°C. Color was developed using 0.02% 3,3′-diaminobenzidine-tetrahydrochloride (Wako Pure Chemical Industries, Ltd.) containing 0.0003% H_2_O_2_ in Tris-buffered saline for 5 min, and sections were then counter-stained with hematoxylin. To provide negative controls, non-immunized rabbit IgG was used instead of primary antibody.

### Statistical analysis

Statistical analyses were carried out using the SPSS Version 17.0 (SPSS, Inc., Chicago, IL, USA) statistics program. All statistical analyses were carried out by the Kruskal–Wallis test and, if significant, by multiple comparisons using the Mann–Whitney *U*-test and SPSS. *p*-values of < 0.05 were considered to be statistically significant.

## Results

### Characterization of anti-TrkB (clone 47/TrkB)

**Fig. 1:**
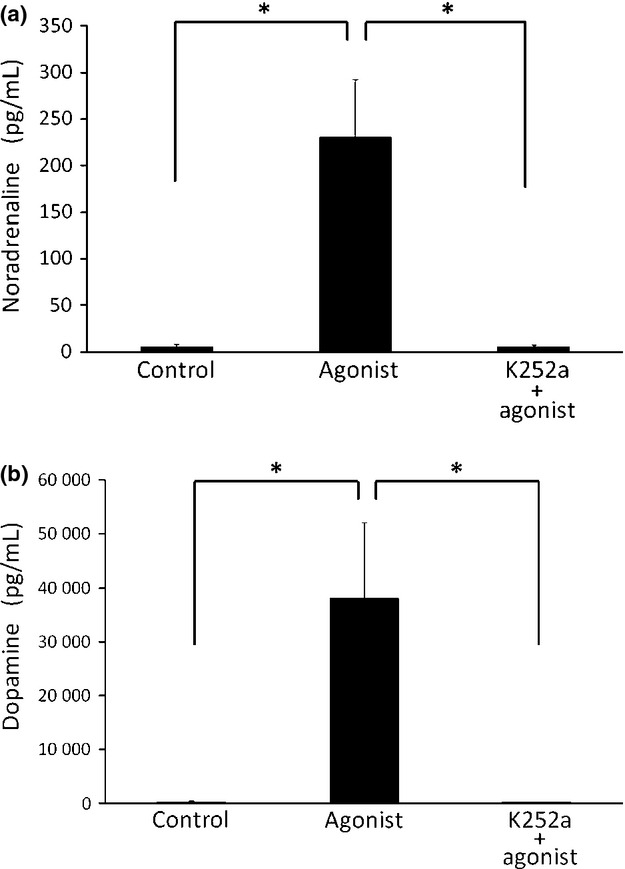
Characterization of anti-tyrosine receptor kinase B (TrkB) (clone 47/TrkB). PC12 cells were treated with anti-TrkB (clone 47/TrkB) and/or TrkB inhibitor, K252a. Levels of noradrenaline (a) and dopamine (b) in the media were then assayed by HPLC. (a) Significant differences in noradrenaline levels were seen between the agonist group and all other groups. Data were analyzed by the Mann–Whitney *U*-test and the Kruskal–Wallis test (*p < 0.05; *n* = 4 per group; error bars = SD). (b) Significant differences in dopamine levels were seen between the agonist group and all other groups. Data were analyzed by the Mann–Whitney *U*-test and the Kruskal–Wallis test. (*p < 0.05; *n* = 4 per group; error bars = SD). Adrenaline was not detected in any group.

Cells were treated with anti-TrkB (clone 47/TrkB) and/or Trk inhibitor K252a, and released catecholamine was purified from the media. Noradrenaline levels in the various groups were as follows: control group, 2.500 ± 1.291 pg/mL; agonist group, 230.8 ± 62.49 pg/mL; and K252a+agonist group, 4.500 ± 3.512 pg/mL. There was a significant difference between the noradrenaline levels in the agonist group and those in all other experimental groups, but there was no significant difference between the K252a+agonist group and the control group (Fig. 1a). Dopamine levels in the various groups were as follows: control group, 373.8 ± 109.2 pg/mL; agonist group, 38087.8 ± 14032.5 pg/mL; and K252a+agonist group, 295.8 ± 84.98 pg/mL. There was a significant difference between the dopamine levels in the agonist group and those in the other experimental groups, but there were no significant differences between the K252a+agonist group and the control group (Fig. 1b). Adrenaline was not detected in the media from any experimental group of cells.

### *In vivo* catecholamine levels after stimulation using TrkB agonist antibody and 60-min stress

**Fig. 2:**
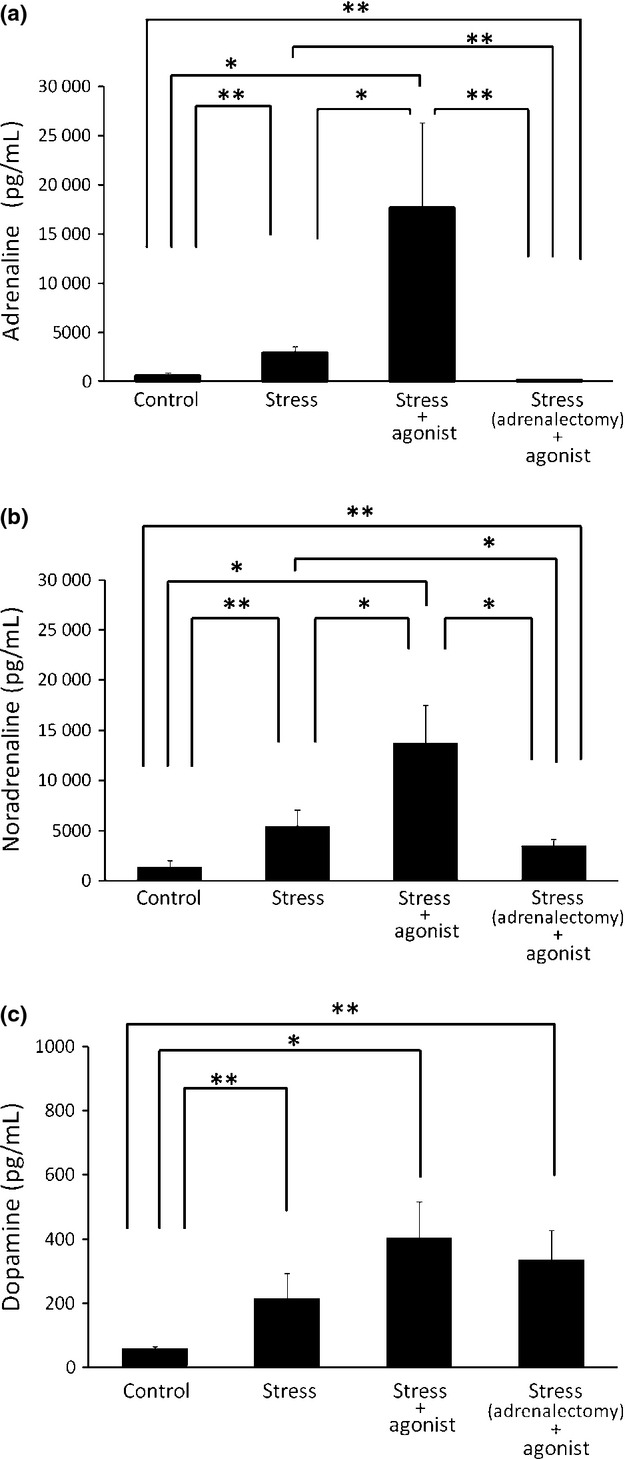
*In vivo* catecholamine levels after stimulation with tyrosine receptor kinase B (TrkB) expressed in the adrenal medulla after 60-min stress using TrkB agonist antibody. Rats were stimulated with TrkB agonist antibody after 60-min immobilization stress. Levels of adrenaline (a), noradrenaline (b) and dopamine (c) in blood were assayed by HPLC. (a) Significant differences in adrenaline levels were seen between each group. Data were analyzed by the Mann–Whitney *U*-test and the Kruskal–Wallis test. (*p < 0.05, **p < 0.01; *n* = 4–6 per group; error bars = SD). (b) Significant differences in noradrenaline levels were seen between each group. Data were analyzed by the Mann–Whitney *U*-test and the Kruskal–Wallis test. (*p < 0.05, **p < 0.01; *n* = 4–6 per group; error bars = SD). (c) Significant differences in dopamine levels were seen between the control group and all other groups, but there were no significant differences between the stress+agonist and stress groups, or between the stress+agonist and stress (adrenalectomy)+agonist groups. Data were analyzed by the Mann–Whitney *U*-test and the Kruskal–Wallis test. (*p < 0.05, **p < 0.01; *n* = 4–6 per group; error bars = SD).

We examined the release of catecholamine in rats subjected to 60 min of TrkB stimulation via TrkB agonist antibody (anti-TrkB; clone 47/TrkB). Adrenaline levels in the various groups were as follows: control group, 431.5 ± 247.6 pg/mL; stress group, 3260.5 ± 462.9 pg/mL; stress+agonist group, 17689.5 ± 8676.9 pg/mL; and stress (adrenalectomy)+agonist group, 2.500 ± 1.300 pg/mL. There were significant differences between the adrenaline levels in each group (Fig. 2a). Noradrenaline levels in the various groups were as follows: control group, 1357.0 ± 677.7 pg/mL; stress group, 6053.8 ± 1585.8 pg/mL; stress+agonist group, 13737.8 ± 3754.9 pg/mL; and stress (adrenalectomy)+agonist group, 3550.8 ± 614.7 pg/mL. Similarly, there were significant differences between the noradrenaline levels in each group (Fig. 2b). Dopamine levels in the various groups were as follows: control group, 61.5 ± 5.2 pg/mL; stress group, 254.0 ± 62.5 pg/mL; stress+agonist group, 405.5 ± 111.0 pg/mL; and stress (adrenalectomy)+agonist group, 338.5 ± 78.5 pg/mL. There were significant differences in dopamine levels between the control group and those in all other groups (Fig. 2c).

In the control, stress and stress+agonist groups, there were no significant differences between intact rats and sham-operated rats. Furthermore, in the stress (adrenalectomy)+agonist group, there were no significant differences between sham-operated rats and stress+agonist group (data not shown).

### Expression of TrkB mRNA and BDNF mRNA in adrenal gland during 60-min stress

**Fig. 3:**
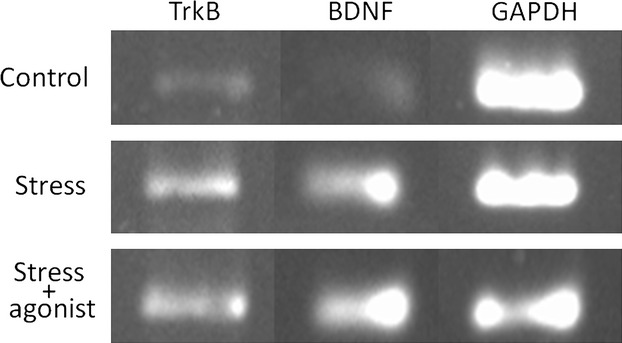
RT-PCR analysis of tyrosine receptor kinase B (TrkB) and Brain-derived neurotrophic factor (BDNF) mRNA expression in adrenal glands after 60-min stress. RT-PCR analysis detected mRNA expression of BDNF and TrkB in the stress and stress+agonist groups. In the control group, very little BDNF and TrkB mRNA expression was observed.

We further analyzed TrkB mRNA and BDNF mRNA expression in the adrenal glands during 60-min immobilization stress using RT-PCR. After 60 min of stress, in the stress and stress+agonist groups, BDNF mRNA was markedly up-regulated in the adrenal gland when compared with the control group. Similarly, TrkB mRNA was weakly up-regulated when compared with the control group (Fig. 3). There were no differences in expression between intact rats and sham-operated rats (data not shown).

### Quantitative real-time PCR analysis of BDNF mRNA in adrenal gland during 60-min stress

**Fig. 4:**
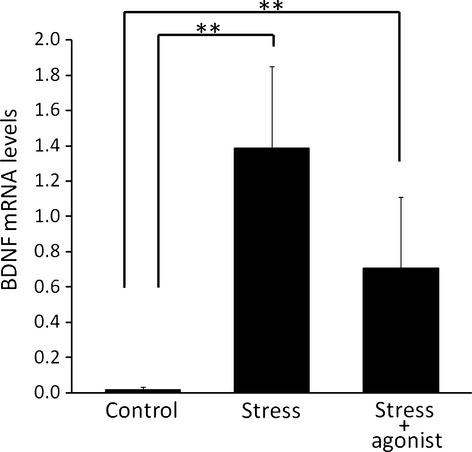
Quantitative real-time PCR analysis of Brain-derived neurotrophic factor (BDNF) mRNA levels in adrenal glands after 60-min stress. BDNF mRNA levels in the adrenal glands of immobilized rats after 60 min was assayed by real-time PCR. Data are expressed as BDNF/β-actin mRNA ratios. BDNF mRNA levels were calculated to be: 0.021 ± 0.017 for the control group (non-stress), 1.254 ± 0.400 for the stress group, and 0.830 ± 0.344 for the stress+agonist group. Significant differences in BDNF mRNA levels were seen between the control group and all other groups. Data were analyzed by the Mann–Whitney *U*-test and the Kruskal–Wallis test (**p < 0.01; *n* = 6 per group; error bars = SD).

Brain-derived neurotrophic factor mRNA levels in the adrenal gland were assayed using real-time PCR. We first confirmed that the PCR product thus obtained was BDNF mRNA. Melting curve analysis of the RT-PCR product revealed a single fluorescent peak representing the Tm (AQ) of BDNF mRNA in all samples other than the negative sample (data not shown). In addition, a single RT-PCR product band was observed following agarose gel electrophoresis (data not shown). BDNF/β-actin mRNA ratios were calculated for the control group, stress group, and stress+agonist group. BDNF/β-actin ratios were as follows: control group, 0.021 ± 0.017; stress group, 1.254 ± 0.400; and stress+agonist group, 0.830 ± 0.344. There was a significant difference between the control group and the other groups. There were no significant differences between the stress group and the stress+agonist group (Fig. 4). There were no significant differences in expression between intact rats and sham-operated rats (data not shown).

### ISH for BDNF mRNA in adrenal gland after 60-min stress

**Fig. 5:**
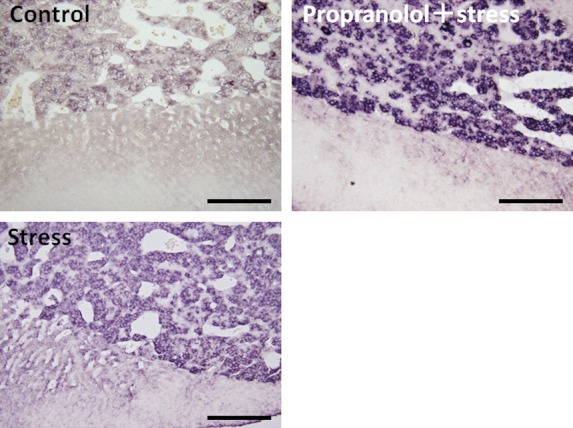
*In situ* hybridization for Brain-derived neurotrophic factor (BDNF) mRNA in adrenal gland after 60-min stress. In the control group, BDNF mRNA was weakly expressed in the adrenal medulla, but was not expressed in the adrenal cortex. In the stress and propranolol+stress groups, BDNF mRNA was expressed in the adrenal medulla, but was not expressed in the adrenal cortex. Scale bar = 200 μm.

We analyzed BDNF mRNA in the adrenal gland after 60-min stress by ISH. A high level of BDNF mRNA expression was detected in positive control rat epididymal cells, but no signals were seen in sections reacted with a sense probe (data not shown). In the control group, hybridization signals were weakly present in adrenal medulla cells, with no signals in adrenal cortex cells. In the stress group, hybridization signals were identified in adrenal medulla cells, with no signals in adrenal cortex cells. Furthermore, in the propranolol+stress group, hybridization signals were identified in adrenal medulla cells, with no signals in adrenal cortex cells (Fig. 5). There were no differences in expression between intact rats and sham-operated rats (data not shown). Sense probes failed to show hybridization signals in these cells (data not shown).

### IHC for BDNF protein in adrenal gland after 60-min stress

**Fig. 6:**
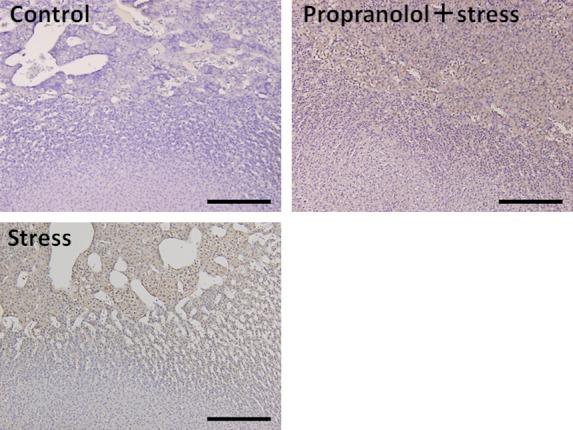
Immunohistochemistry for Brain-derived neurotrophic factor (BDNF) protein in adrenal gland after 60-min stress. In the control group, BDNF protein was weakly detected in the adrenal medulla, but was not detected in the adrenal cortex. In the stress and propranolol+stress groups, BDNF protein was detected in the adrenal medulla, but was not detected in the adrenal cortex. Scale bar = 200 μm.

In the control group, BDNF protein was weakly detected in the adrenal medulla, but was not detected in the adrenal cortex. In the stress group, BDNF protein was detected in the adrenal medulla, but was not detected in the adrenal cortex. Similarly, in the propranolol+stress group, BDNF protein was detected in the adrenal medulla, but was not detected in the adrenal cortex (Fig. 6). There were no differences in expression between intact rats and sham-operated rats (data not shown). Detection of BDNF protein in the stressed adrenal medulla was abolished by competition with recombinant BDNF (data not shown).

## Discussion

We first investigated whether anti-TrkB (clone 47/TrkB) can act as a TrkB agonist antibody, and found that significant levels of catecholamines were released from PC12 cells (Fig. 1a and b). In addition, catecholamine release was completely blocked by the tyrosine kinase inhibitor K252a. These results show that anti-TrkB (clone 47/TrkB) is able to act as a TrkB agonist antibody, and these results were similar to those obtained with BDNF stimulation (Kondo *et al*. 2010). In this experiment, adrenaline was below the detection limit. As adrenaline is synthesized by noradrenaline methylation with phenylethanolamine–*N*-methyltransferase, and activation of phenylethanolamine–*N*-methyltransferase is essential for the presence of adrenal cortical hormone (Wurtman and Axelrod 1966). We experimented with chromaffin cells (no adrenal cortical cells present) *in vitro* and were unable to detect adrenaline. Qian *et al*. (2006)reported multiple mouse monoclonal antibodies function as a TrkB agonist antibody. In addition, Tsao *et al*. (2008) used agonist antibody to exert anti-TrkB activity *in vivo*. Thus, we established that the use of anti-TrkB (clone 47/TrkB) as a TrkB agonist antibody was appropriate.

In a previous study, TrkB mRNA and protein expression were significantly up-regulated in the adrenal medulla under acute immobilization stress for 60 min in rats (Kondo *et al*. 2010). This confirmed that TrkB of expression in the adrenal medulla could play an important role in stress response. We then examined the release of catecholamine after 60-min stress using TrkB agonist antibody (anti-TrkB; clone 47/TrkB). Groups of rats stimulated with agonist antibody demonstrated significant increases in blood catecholamine levels, as compared with unstimulated rats, but these levels did not increase in adrenalectomized model rats (adrenalectomized rats were stimulated with agonist after 60-min stress) (Fig. 2a–c). These data suggest that signal transduction by TrkB in the adrenal medulla evokes catecholamine release.

In adrenalectomized model rats, catecholamine levels were low (Fig. 2a–c). Blood catecholamine is mainly derived from the adrenal medulla and is released from the adrenal medulla, as well as various other organs (Nakata 1964). These results were considered to be derived from organs other than the adrenal medulla.

Brain-derived neurotrophic factor mRNA was detected in the adrenal gland after 60-min stress using PCR (Figs 3 and 4). These results showed that expression of BDNF and TrkB (Kondo *et al*. 2010) in the adrenal gland after 60-min stress induces catecholamine release.

We used ISH and IHC to investigate the localization of BDNF expressed in the adrenal gland after 60-min stress. BDNF was localized in the adrenal medulla (Figs 5 and 6), which suggests that the BDNF and TrkB expressed in the adrenal medulla during 60-min stress evoked catecholamine release. In addition, catecholamine release was evoked by activation of nicotine receptor via SAM axis, as well as autocrine signaling by BDNF in the adrenal medulla. Meanwhile, Tsukinoki *et al*. (2007) suggested that BDNF is expressed after 60-min immobilization stress in submandibular glands of rats, and that plasma BDNF contributes to BDNF expression in the submandibular gland. Therefore, our results must consider the presence of BDNF derived from sources other than the adrenal medulla. With respect to autocrine signaling in the adrenal medulla, Cortez *et al*. (2012) suggested that beta-adrenoceptors are expressed in the adrenal medulla, and that these regulated catecholamine release with autocrine signaling. Thus, catecholamine release via autocrine BDNF–TrkB interactions appears to function in the adrenal medulla.

In the adrenal glands of rats exposed to 60-min stress after administration of propranolol, BDNF was localized to the adrenal medulla (Figs 5 and 6). This suggests that BDNF–TrkB interaction plays a role in a positive feedback loop in the adrenal medulla. However, from neurotransmission and synaptic transmission viewpoints, further studies are necessary to confirm the mechanisms of this positive feedback loop. Positive feedback loops remain known to exist in the homeostatic mechanisms, for example, aldosterone release in the adrenal cortex and enteric nervous system (Gallo-Payet *et al*. 1996; Bertrand and Thomas 2004). In addition, Cortez *et al*. (2012)suggested that beta-adrenoceptors expressed in the adrenal medulla regulate catecholamine release via a positive feedback loop. Therefore, the adrenal medulla may play a role in the positive feedback loop of BDNF–TrkB interaction. Higo *et al*. (2010)also suggested that severe stress induces endoplasmic reticulum stress in neuronal cell, leading to cell death. This would probably result in breakdown of the SAM axis. To prevent these events, a positive feedback loop may act as an alternative pathway to the SAM axis when severe stress induces nerve damage.

TrkB has three major signaling cascades: the phospholipase C-γ (PLC-γ) pathway; the phosphatidylinositol-3-kinase (PI3-K) and Akt-kinase pathways; and the Ras-MAPK pathway (Rose *et al*. 2004). In particular, the PLC-γ pathway induces calcium release from intracellular stores by activating inositol 1,4,5-trisphosphate (IP3) (Rose *et al*. 2004). Therefore, we suspected exocytosis of catecholamine because of the increase in intracellular calcium concentration via the PLC-γ pathway activated by BDNF–TrkB interaction. Stress response is regulated by multiple mechanisms *in vivo* (Kvetnansky *et al*. 2009). In the adrenal medulla, BDNF is likely to be a key player during stress, and may regulate expression of several genes and neuropeptides (Liu *et al*. 2008). Therefore, BDNF expressed in the adrenal medulla can play various roles in stress response other than catecholamine release. Further studies are necessary to fully elucidate the signaling pathway and its role in stress response.

In conclusion, we demonstrated that anti-TrkB (clone 47/TrkB) acts as a TrkB agonist antibody. Furthermore, this study suggests for the first time that TrkB in the adrenal medulla can evoke catecholamine release in rats under 60-min stress conditions using TrkB agonist antibody, and BDNF expression was confirmed in the adrenal medulla of these rats. Therefore, BDNF may have an autocrine function in the adrenal medulla. Finally, the adrenal medulla may play a role in a positive feedback loop through autocrine BDNF–TrkB interactions under acute stress conditions.

## Abbreviations

BDNF: brain-derived neurotrophic factor
cDNA: complementary DNA
cRNA: complementary RNA
DAB: 3,3′-diaminobenzidine-tetrahydrochloride
DIG: digoxigenin
GAPDH: glyceraldehyde-3-phosphate dehydrogenase
HPA axis: hypothalamic–pituitary–adrenal axis
HRP: horseradish peroxidase
i.p.: intraperitoneally
IHC: immunohistochemistry
IP3: inositol 1,4,5-trisphosphate
ISH: *in situ* hybridization
MAPK: mitogen-activated protein kinase
NGF: nerve growth factor
p75: p75 neurotrophin receptor
PI3-K: phosphatidylinositol-3-kinase
PLC-γ: phospholipase C-γ
SAM axis: sympathetic–adrenal–medullary axis
TBS: tris-buffered saline
TrkB: tyrosine receptor kinase B
